# Mitochondrial neurogastrointestinal encephalomyopathy in a Pakistani female: a case report 

**DOI:** 10.1186/s13256-022-03582-6

**Published:** 2022-10-03

**Authors:** Zaraq Rashid Khan, Alvina Karam, Mian Ayaz ul Haq, Aleena Aman, Ahmad Sharjeel Karam

**Affiliations:** 1 Hayatabad Medical Complex, Peshawar, Pakistan; 2 Shaukat Khanum Memorial Cancer Hospital, Peshawar, Pakistan; 3 Gandhara University Kabir Medical College, Peshawar, Pakistan

**Keywords:** MNGIE, Asymptomatic leukoencephalopathy, Mitochondrial disorders, Genetic studies, Gastroparesis

## Abstract

**Background:**

Mitochondrial neurogastrointestinal encephalopathy is a rare multisystem autosomal recessive disease caused by mutations in the *TYMP* gene, that encodes for thymidine phosphorylase. Mitochondrial neurogastrointestinal encephalopathy is a progressive degenerative disease characterized by a distinctive tetrad of gastrointestinal dysmotility, peripheral neuropathy, ophthalmoplegia with ptosis, and asymptomatic leukoencephalopathy. It provides a diagnostic dilemma to physicians in regions like Pakistan because of a lack of genetic study availability and associated financial constraints of the population. However, with careful examination and a few basic investigations, mitochondrial neurogastrointestinal encephalopathy can be diagnosed by ruling out most of the close differentials.

**Case presentation:**

We report the case of a 23-year-old Asian female whose chief complaints were epigastric pain, bilious emesis, weight loss for 3 months, and bilateral lower limb weakness for 20 days. All clinical signs and symptoms along with relevant investigations including nerve conduction studies, electromyography, and magnetic resonance imaging of the brain were highly suggestive of mitochondrial neurogastrointestinal encephalopathy syndrome. Because of financial constraints, genetic studies could not be performed. The patient was managed with a multidisciplinary approach involving gastroenterology, physiotherapy, and nutrition departments. Currently, therapeutic options for the disease include allogeneic hematopoietic stem cell transplant and carrier erythrocyte entrapped thymidine phosphorylase; however, these could not be provided to the patient owing to certain limitations.

**Conclusions:**

As misdiagnosis and delayed diagnosis are quite common in this disease, the prime objective of this case report is to increase the basic understanding of this disease, especially its signs and symptoms, and address the limitations regarding the diagnostic investigations and management of patients with mitochondrial neurogastrointestinal encephalopathy.

## Introduction

Mitochondrial neurogastrointestinal encephalopathy (MNGIE) is an autosomal recessive disorder caused by mutations in the *TYMP* gene, which encodes for thymidine phosphorylase [1]. Our search revealed more than 80 pathogenic mutations in the *TYMP* gene correlated with the disorder, documented in patients from multiple ethnic populations [2]. Earlier studies have inferred that these mutations cause an absolute or near-approximate loss of the activity of thymidine phosphorylase, generating toxic accumulations of nucleoside thymidine and deoxyuridine in tissues [2–4]. The aggregation of these nucleotides is crucial for the instability of mitochondrial DNA (mtDNA) and the consequence of the phenotypic and molecular aberrations noted in this disease [5]. The prevalence of MNGIE is approximately 1–9 per 1,000,000 [6].

MNGIE is characterized by severe progressive gastrointestinal dysmotility, leukoencephalopathy, peripheral neuropathy, and ocular symptoms [1]. The symptoms generally start in the second or third decade of life (the average age at onset is 19 years) [7], whereas a sharp deterioration in survival is seen in the fourth decade of life [1, 2]. Various treatment options have been proposed for MNGIE syndrome with variable success, including allogeneic hematopoietic stem cell transplant, gene therapy, enzyme replacement therapy, and orthotropic liver transplantation [8]. Here we present a case report of MNGIE syndrome in a 23-year-old Asian girl who presented to our department with symptoms of gastroparesis, ophthalmoplegia, cachexia, and peripheral sensorimotor neuropathy. Written informed consent was obtained from the patient.

## Case presentation

A 23-year-old Asian female, with no previous comorbidities, presented to us with a 3-month history of nonradiating postprandial epigastric pain, bilious emesis, and weight loss. She also complained of having bilateral lower limb weakness that started 20 days back. Initially, the patient showed a good response to prokinetic drugs and changes in the diet, but gradually her condition deteriorated. She recorded a 20 kg weight loss.

On arrival at our department, the patient was hemodynamically stable. On physical examination, she appeared cachectic and had a burn injury scar on her face. Her abdominal examination and cardiovascular examination were normal. On neurological examination, she was well oriented in time, place, person, and perception. She had decreased tone in bilateral lower limbs with a power of 2/5. She had absent reflexes with downgoing plantars in bilateral lower limbs. Her sensory examination of lower limbs revealed diminished vibratory and proprioception sensations; however, pain, temperature, and touch sensations were intact. The rest of her central nervous system examination was normal. Additionally, an ophthalmologist was taken on board as she had limitations in gaze across all four quadrants consistent with complex ophthalmoplegia. The rest of the ophthalmic examination was normal.

**Fig. 1:**
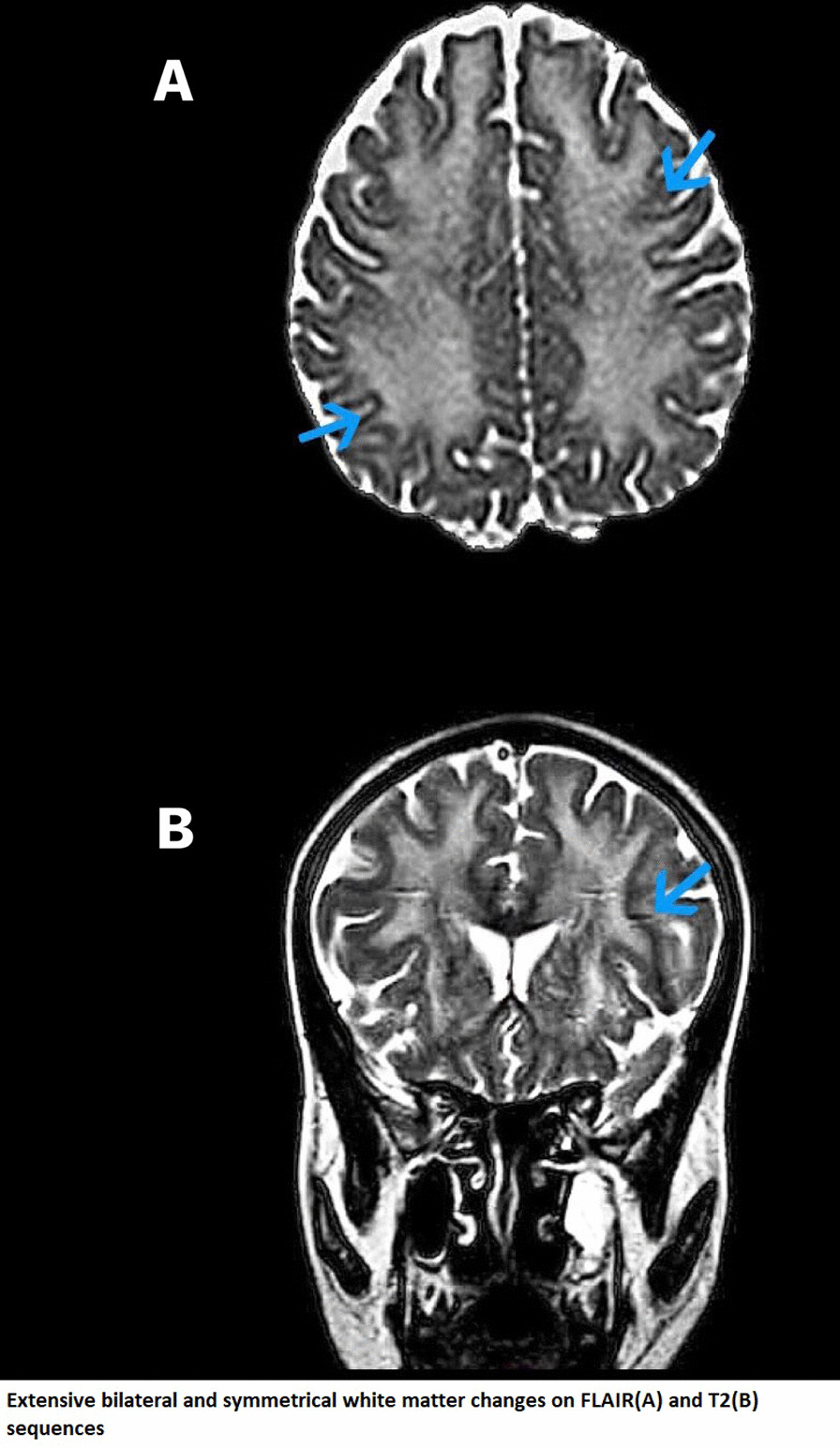
Magnetic resonance imaging brain showing T2 and flair sequences. The arrow points out to demyelinating white matter changes in T2 and flair sequences

Previously, the patient had undergone laboratory workup and imaging studies to investigate her gastrointestinal symptoms. All the investigations done, including a computed tomography (CT) scan of the abdomen as well as pelvis, duodenal, and jejunal mucosal biopsies, were unremarkable except for upper gastrointestinal (GI) endoscopy, which revealed reflux esophagitis. Her stool routine examination was unremarkable. A review of the patient’s past record did not reveal the procedure used to obtain the aforementioned biopsies. Her celiac serology was negative as well. Furthermore, her gastroparesis was confirmed with gastric emptying scintigraphy. Her repeated complete blood profile and smear showed blood hemoglobin of 10.7 g/dL with a microcytic and hypochromic picture. The rest of her investigations including basal metabolic panel, hemoglobin A1c, and lipid profile were normal (Table 1). Nerve conduction studies (NCS) revealed peripheral sensorimotor neuropathy, demyelinating in type with secondary axonal degeneration. Electromyography (EMG) was advised, which revealed myopathic discharges in proximal as well as distal muscle groups, most prominent in lower limbs. Magnetic resonance imaging (MRI) of the brain revealed extensive lesions suggestive of leukoencephalopathy (Fig. 1).

**Table 1:** Metabolic profile of the patient

Investigations	Patient value	Reference value
White blood cell count	9.7 × 10^3^/mm^3^	4.5–11.0 × 10^3^/mm^3^
Hemoglobin	10.7 g/dL	12.0–16.0 g/dL
Red blood cell count	3.9 × 10^6^/µL	3.9–5.2 × 10^6^/µL
Platelets (thrombocytes)	350 × 10^3^/µL	130–400 × 10^3^/µL
Mean corpuscular volume	77 µm^3^	78–102 µm^3^
Erythrocyte sedimentation rate	28 mm/hour	30 mm/hour
C-reactive protein	< 0.5 mg/dL	< 0.5 mg/dL
Creatine kinase	30 units/L	0–160 units/L
Alanine aminotransferase	14 units/L	7–30 units/L
Alkaline phosphatase	56 units/L	30–100 units/L
Aspartate aminotransferase	20 units/L	9–25 units/L
Hemoglobin A1c	4.0%	< 6.5%
Cholesterol	150 mg/dL	< 200 mg/dL
Low-density lipoprotein	40 mg/dL	< 100 mg/dL
High-density lipoprotein	65 mg/dL	> 60 mg/dL
Triglycerides	50 mg/dL	40–150 mg/dL
Lactic acid	2.0 mmol/L	0.5–2.2 mmol/L
Bilirubin—total	1.0 mg/dL	0.0–1.0 mg/dL
Sodium	136 mmol/L	135–150 mmol/L
Potassium	3.6 mmol/L	3.5–5.1 mmol/L
Albumin, serum	3.3 g/dL	3.1–4.3 g/dL
Chloride	98 mmol/L	95–108 mmol/L
Calcium, serum	8.2 mg/dL	8.0–10.5 mg/dL
Glucose, random	72 mg/dL	70–140 mg/dL

On the basis of the classic clinical signs and symptoms observed along with the imaging studies, MNGIE syndrome was suspected and the patient was advised to undergo genetic testing and blood nucleoside studies. These investigations, however, were not possible. Owing to financial constraints and limited laboratory genetic testing capabilities in our setup, the patient’s plasma thymidine level was not assessed. Nevertheless, in the light of the strong clinical evidence, the patient was diagnosed with MNGIE syndrome.

The patient was managed with a multidisciplinary approach including a gastroenterologist, a specialist nutritionist, and a physiotherapist. She was advised on total parenteral nutrition, which would improve her nutritional status and act as a bridge therapy until a thorough evaluation for a possible jejunostomy tube is done. Thorough counseling of the patient and her family was done regarding the nature and pathology of the disease including the medications she was to avoid. The need for genetic testing of the patient and other family members was reemphasized. However, they denied having further investigations owing to a scarcity of funds.

The patient was managed with an injection of metoclopramide 10 mg intravenously thrice daily, an injection of omeprazole 40 mg intravenously once daily, a tablet of coenzyme Q10 50 mg once daily, and a daily dose of a multivitamin.

Various treatment options, including a possible allogeneic hematopoietic stem cell transplant and liver transplantation, were also discussed with the family. The patient was then referred to the gastroenterology department for a jejunostomy tube; however, the patient decided against the advice. She was discharged on tablet metoclopramide 10 mg thrice daily before meal, tablet omeprazole 40 mg once daily before breakfast, tablet coenzyme Q10 50 mg once daily, and multivitamins. She was advised to maintain strict compliance to nutritional and physiotherapy recommendations and to follow up in 1 month. Unfortunately, the patient was lost to follow-up despite multiple attempts to contact the patient or her family members.

## Discussion

MNGIE is an autosomal recessive disorder that is difficult to interpret and often goes undiagnosed. The pathophysiology of MNGIE includes mitochondrial dysfunction caused by a modification in the *TYMP* gene. The *TYMP* gene codes for the thymidine phosphorylase (TP) and is situated on chromosome 22 [1]. It is a rare syndrome with high mortality and inadequate treatment modalities available.

Our patient was a 23-year-old woman who presented with postprandial epigastric pain, bilious emesis, and weight loss. These clinical features were consistent with other gastrointestinal manifestations of MNGIE (for example, satiety, vomiting, abdominal pain, constipation, cramps, diarrhea, borborygmi, or pseudo-obstruction) leading to weight loss and cachexia. Neurological manifestations were seen in our patient as she complained of having bilateral lower limb weakness. The most frequent neurological symptoms of MNGIE are peripheral neuropathy, ptosis, leukoencephalopathy, hearing loss, and ophthalmoparesis [7, 9]. Neuropathy in MNGIE arises later but is prevalent and oftentimes debilitating.

The constellation of symptoms such as leukoencephalopathy and peripheral neuropathy in a young adult narrows the differential significantly, but these symptoms are often surpassed by the substantial GI manifestations and the rate of initial misdiagnosis is huge. It is frequently misdiagnosed as anorexia nervosa, inflammatory bowel disease (IBD), malabsorption syndrome, or intestinal pseudo-obstruction and is unnecessarily treated with surgery. This delays the diagnosis by up to 10 years as reported in the literature [10].

MNGIE is a rare disorder with a poor prognosis, and awareness of this disorder is highly crucial. The clinical diagnosis is based on the presence of major symptoms or by detection of mutation in *TYMP*, decreased TP activity, or elevated nucleosides. Diagnosis of MNGIE relies on two major criteria: (1) exclusion of acquired causes, and (2) assessment of the constellation of symptoms, encouraging confirmatory testing. Our patient had similar clinical and imaging results seen in MNGIE. However, owing to inadequate funding and limited laboratory testing capabilities in our setup, genetic workup was not performed.

Contemporary treatment modalities for MNGIE primarily emphasize restoring the activity of TP and reducing the levels of the nucleosides, hematopoietic stem cell replacement (HSCT), liver transplantation, or gene therapy [10]. Dialysis can also temporarily reduce the level of circulating nucleosides [11, 12]. Hematopoietic stem cell transplant (HSCT) is the best-studied treatment regimen and has been confirmed to provide clinical improvement. Halter *et al*. documented that retrospective research on 24 patients who had HSCT for MNGIE syndrome revealed a survival rate of 37.5% after an average follow-up of almost 4 years [13]. Farahvash *et al*. reported that, among 102 patients with MNGIE, the median age of death was 35 years (range 14–54 years) [14].

Our patient was managed with a multidisciplinary approach comprising a gastroenterologist, a specialist nutritionist, and a physiotherapist focusing on the nutritional status of the patient.

The designation of a biomarker for MNGIE would lead to improved patient outcomes through expediting patient access to novel treatments, facilitating timely intervention, and assessment of therapy efficacy. One of the proposals of the latest published outcome from the International Consensus Conference on MNGIE was the use of multi-omics analyses to specify biomarkers that fingerprint the major clinical symptoms of the disorder [15].

Our case is noteworthy because it accentuates a rare cause of gastrointestinal manifestations and diagnoses in a less resourceful setup. Many patients are repeatedly misdiagnosed owing to the nonspecificity of clinical features. Consequently, this issue further stresses that physicians should be observant in comprehending rare diseases presenting with GI dysmotility and neuropathy, especially in young adults, to ensure a timely intervention.

## Conclusion

MNGIE is a rare clinical entity, and as such there are many cases of delayed diagnosis reported in various setups. The objective of this study was to report a case of MNGIE syndrome in a setup where the diagnostic investigations specific to MNGIE are not available and physicians hence rely heavily on classical clinical signs and symptoms along with basic investigations. Additionally, we hope to create much-needed awareness regarding the disease, so that it can be diagnosed early and patients receive optimum treatment. Case reports like this will also open the door to discussions regarding the need for advanced clinical labs in developing countries like Pakistan.

## Data Availability

Data sharing does not apply to this article as no datasets were generated or analyzed during the current study.
